# Prognostic Impact of MiR-155 in Non-Small Cell Lung Cancer Evaluated by *in Situ *Hybridization

**DOI:** 10.1186/1479-5876-9-6

**Published:** 2011-01-10

**Authors:** Tom Donnem, Katrine Eklo, Thomas Berg, Sveinung W Sorbye, Kenneth Lonvik, Samer Al-Saad, Khalid Al-Shibli, Sigve Andersen, Helge Stenvold, Roy M Bremnes, Lill-Tove Busund

**Affiliations:** 1 Department of Oncology, University Hospital of North Norway, Tromso, Norway; 2 Institute of Clinical Medicine, University of Tromso, Tromso, Norway; 3 Department of Pathology, University Hospital of North Norway, Tromso, Norway; 4 Institute of Medical Biology, University of Tromso, Tromso, Norway; 5 Department of Pathology, Nordland Central Hospital, Bodo, Norway

## Abstract

**Background:**

In recent years, microRNAs (miRNAs) have been found to play an essential role in tumor development. In lung tumorigenesis, targets and pathways of miRNAs are being revealed, and further translational research in this field is warranted. MiR-155 is one of the miRNAs most consistently involved in various neoplastic diseases. We aimed to investigate the prognostic impact of the multifunctional miR-155 in non-small cell lung cancer (NSCLC) patients.

**Methods:**

Tumor tissue samples from 335 resected stage I to IIIA NSCLC patients were obtained and tissue microarrays (TMAs) were constructed with four cores from each tumor specimen. *In situ *hybridization (ISH) was used to evaluate the expression of miR-155.

**Results:**

There were 191 squamous cell carcinomas (SCCs), 95 adenocarcinomas (ACs), 31 large cell carcinomas and 18 bronchioalveolar carcinomas. MiR-155 expression did not have a significant prognostic impact in the total cohort (P = 0.43). In ACs, high miR-155 expression tended to a significant negative prognostic effect on survival in univariate analysis (P = 0.086) and was an independent prognostic factor in multivariate analysis (HR 1.87, CI 95% 1.01 - 3.48, P = 0.047). In SCC patients with lymph node metastasis, however, miR-155 had a positive prognostic impact on survival in univariate (P = 0.034) as well as in multivariate (HR 0.45, CI 95% 0.21-0.96, P = 0.039) analysis.

**Conclusions:**

The prognostic impact of miR-155 depends on histological subtype and nodal status in NSCLC.

## Introduction

Lung cancer is the leading cause of cancer-related mortality in both men and women [1]. Despite several new treatment achievements, the consistently poor 5-year survival for lung cancer patients underscores the need for novel modalities for early detection, prognostification and targeted therapies [1,2].

MicroRNAs (miRNAs) are approximately 19-22 nucleotides single stranded RNAs playing crucial roles in regulating gene expression by either inducing mRNA degradation or inhibiting translation [3,4]. These non-coding RNAs can simultaneously regulate hundreds to thousands of their target genes or up to one third of the genome, thereby controlling a wide range of biological functions including apoptosis, proliferation and differentiation [3,5].

To date miR-155 is one of the miRNAs most consistently involved in neoplastic diseases in both hematopoietic malignancies (i.e. Hodgkin's lymphoma, some types of Non Hodgkin's lymphoma, AML and CML) and solid tumors (e.g. breast, colon, cervical, thyroid, pancreatic and lung cancer) [6-16]. MiR-155 is also involved in other biological processes like hematopoiesis, inflammation and immunity [6]. The frequently detected up-regulation of miR-155 in malignant cells indicates a major role as an oncogene, however, a possible tumor suppression function has also been suggested [17]. In non-small cell lung cancer (NSCLC), miR-155 has so far been considered as an oncogene and been associated with a poor prognosis [13,16], though a recent large scale study did not find miR-155 to have any prognostic or predictive impact [18].

NSCLC classification according to histology and nodal status are two of the most important determinants for NSCLC treatment strategies [13,19]. However, a considerable variability in prognosis has been observed for subsets of patients with the same clinical features. Consequently, the clinical incorporation of predictive and prognostic molecular biomarkers with traditional cancer staging should improve the management of patients with NSCLC.

Squamous cell carcinomas (SCCs) and adenocarcinomas (ACs) are the major histological subtypes of NSCLC. During recent years, treatment responses and side effects by novel therapies have been correlated to NSCLC subgroups according to histology, gender, ethnicity and smoking status. The vascular endothelial growth factor (VEGF) monoclonal antibody, bevacizumab, is only given to non-SCCs due to the risk of fatal bleeding in SCCs [20]. Further, mutations in epidermal growth factor receptor (EGFR) and response to EGFR tyrosine kinase inhibitors appear related to ACs, female gender, Asian ethnicity and non-smokers, and the new antifolate agent pemetrexed appears to have better response in non-SCC patients and females [21,22]. Consequently, ACs and SCCs are increasingly recognized as different diseases instead of one.

In an unselected NSCLC cohort of 335 patients [23] we aimed to explore, using *in situ *hybridization on a high throughput platform, possible prognostic roles by miR-155 in all NSCLC cases and subgroups according to histology and stage.

## Patients and Methods

### Patients and Clinical Samples

Primary tumor tissues from anonymized patients diagnosed with NSCLC pathologic stage I to IIIA at the University Hospital of Northern Norway (UNN) and Nordland Central Hospital (NLCH) from 1990 through 2004 were used in this retrospective study. In total, 371 patients were registered from the hospital database. Of these, 36 patients were excluded from the study due to: (i) Radiotherapy or chemotherapy prior to surgery (n = 10); (ii) Other malignancy within five years prior to NSCLC diagnosis (n = 13); (iii) Inadequate paraffin-embedded fixed tissue blocks (n = 13). Adjuvant chemotherapy was not introduced in Norway during this period (1990 - 2004). Thus, 335 patients with complete medical records and adequate paraffin-embedded tissue blocks were eligible.

This report includes follow-up data as of November 30, 2008. The median follow-up of survivors was 86 (range 48-216) months. The tumors were staged according to the new 7th edition of TNM in Lung Cancer and histologically subtyped and graded according to the World Health Organization guidelines [19,24]. Regarding N-status, ipsilateral peribronchial or hilar nodes and intrapulmonary nodes are defined as N1, while N2 includes ipsilateral mediastinal or subcarinal nodes. The term N+ (lymph node metastasis present) includes both N1 and N2. The National Data Inspection Board and The Regional Committee for Research Ethics approved the study.

### Microarray Construction

All lung cancer cases were histologically reviewed by two pathologists (S.A.S. and K.A.S.) and the most representative areas of viable tumor cells were carefully selected. The TMAs were assembled using a tissue-arraying instrument (Beecher Instruments, Silver Springs, MD). The Detailed methodology has been previously reported [23]. Briefly, we used a 0.6 mm diameter stylet, and the study specimens were routinely sampled with four replicate core samples (different areas) of tumor tissue. In addition normal lung tissue localized distant from the primary tumor, and one slide with normal lung tissue samples from 20 patients without a cancer diagnosis were stained. Multiple 4-μm sections were cut with a Micron microtome (HM355S) and used for in situ hybridization analysis.

### In Situ Hybridization (ISH)

In situ hybridization was performed following the protocol developed by Nuovo et al.[25], with some minor adjustments. Digoxigenin (DIG) labeled locked nucleic acid (LNA) modified probes for miR-155 (hsa-miR-155), positive control (U6, hsa/mmu/rno) and negative control (scramble-miR) were purchased from Exiqon, Vedbek, Denmark.

Briefly, we placed 4 μm sections of the TMA blocks in a heater at 59°C over night to attach cores to the silane-coated slide. Sections were deparaffinised with xylene (2 × 5 min), rehydrated with ethanol (100 - 50 - 25% for 5 min each), and treated with DEPC water for 1 min. Protease treatment was performed with pepsin solution (1.3 mg/ml) (Dako, Glostrup, Denmark) at 37°C for 50 min. Following a postfixation step in 4% paraformaldehyde (PFA), hybridization of the LNA-probe was carried out in a Hybrite (Abbott Laboratories, IL) at 60°C for 5 min and 37°C over night (12-18 h). Low-stringency post-hybridization wash done at 4°C in SSC with 2% BSA for 5 min, followed by incubation with anti-DIG/alkaline phosphate conjugate antibodies (Enzo Diagnostics, NY) in a heater at 37°C for 30 min. The blue color was developed by incubation of the slide with nitroblue tetrazolium and bromchloroindolyl phosphate (NBT/BCIP) (Enzo Diagnostics, NY) at 37°C. The colorimetric reaction was monitored visually and stopped by placing the slides in water when background coloring started to appear on the negative control (scrambled probe), varying from 15-30 min. The slides were counterstained with nuclear fast red (Enzo Diagnostics, NY) to visualize the nuclei, before cover glass mounting.

### Scoring of ISH

**Figure 1:**
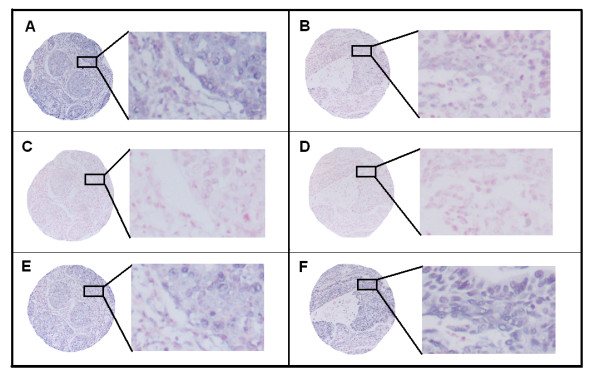
***In situ *hybridization (ISH) analysis of NSCLC representing strong and weak intensities for tumor cell miR-155 expression**. Negative (scramble-miR) and positive (U6) controls from the same tissue area are shown. Strong miR-155 staining (A) with corresponding negative (C) and positive (E) controls to the left. Weak miR-155 staining (B) with corresponding negative (D) and positive (F) controls to the right. ISH positive signals (miR-155 and U6) stain blue, while nuclei stain red.

The ARIOL imaging system (Genetix, San Jose, CA) was used to scan the TMA slides of ISH staining. The slides were loaded in the automated loader (Applied Imaging SL 50) and specimens were scanned at low (1.25×) and high resolution (20×) using the Olympus BX 61 microscope with an automated platform (Prior). Representative and viable tissue sections were scored manually semiquantitatively for cytoplasmic staining on computer screen. The dominant staining intensity in tumor cells was scored as: 0 = negative; 1 = weak; 2 = intermediate; 3 = strong (Figure 1). In case of disagreement (score discrepancy >1), the slides were re-examined and a consensus was reached by the observers. In most cores there was a mixture of stromal cells and tumor cells. By morphological criteria only tumor cells were scored staining intensity.

All samples were anonymized and independently scored by one experienced pathologist and one technician (S.W.S. and K.E.). When assessing a variable for a given core, the observers were blinded to the scores of the other observer and to outcome. Mean score for each case was calculated from all four cores and both examiners. The median miR-155 expression value was used as cut-off.

### Statistics

All statistical analyses were done using the statistical package SPSS (Chicago, IL), version 17. The Chi-square test and Fishers Exact test were used to examine the association between molecular marker expression and various clinicopathological parameters. The ISH scores from each observer were compared for interobserver variability by use of a two-way random effect model with absolute agreement definition. The intraclass correlation coefficient (reliability coefficient) was obtained from these results. Plots of the disease-specific survival (DSS) according to marker expression were drawn using Kaplan-Meier method, and statistical significance between survival curves was assessed by the log rank test. DSS was determined from the date of surgery to the time of lung cancer death. The multivariate analysis was carried out using the Cox proportional hazards model. Variables with P < 0.1 from the univariate analysis were entered into the Cox regression analysis. The significance level used was P < 0.05.

## Results

### Clinicopathological Variables

Demographic, clinical, and histopathological variables are shown in Table 1. The median age was 67 (range, 28-85) years and the majority of patients were male (75%). The NSCLC tumors comprised 191 squamous cell carcinomas (SCCs), 95 adenocarcinomas (ACs), 31 large cell carcinomas and 18 bronchioloalveolar carcinomas. Due to nodal metastasis or non-radical surgical margins, 59 (18%) patients received adjuvant radiotherapy.

**Table 1:** Prognostic Clinicopathologic Variables as Predictors for Disease-Specific Survival in 335 NSCLC Patients (Univariate Analyses; Log-rank Test).

Characteristic	Patients (n)	Patients (%)	Median survival (months)	5-Year survival (%)	P
**Age**					0.34
≤65 years	156	47	83	55	
>65 years	179	53	NR	60	
**Sex**					0.20
Female	82	25	190	63	
Male	253	75	83	56	
**Smoking**					0.23
Never	15	5	19	43	
Current	215	64	NR	60	
Former	105	31	71	54	
**Performance status**					**0.013**
ECOG 0	197	59	NR	63	
ECOG 1	120	36	64	52	
ECOG 2	18	5	25	33	
**Weight loss**					0.71
<10%	303	90	127	58	
>10%	32	10	98	57	
**Histology**					**0.028**
SCC	191	57	NR	66	
Adenocarcinoma	95	34	47	41	
LCC	31	9	98	56	
BAC	18		NR	71	
**Differentiation**					**<0.001**
Poor	138	41	47	47	
Moderate	144	43	190	64	
Well	53	16	NR	68	
**Surgical procedure**					**0.004**
Lobectomy + Wedge*	243	73	190	61	
Pneumonectomy	92	27	37	47	
**Pathological stage**					**<0.001**
I	157	47	190	71	
II	136	41	61	51	
IIIa	42	12	17	23	
**Tumor status**					**<0.001**
1	85	25	190	74	
2	188	56	84	57	
3	62	19	25	36	
**Nodal status**					**<0.001**
0	232	69	190	66	
1	76	23	35	43	
2	27	8	18	18	
**Surgical margins**					0.29
Free	307	92	190	58	
Not free	28	8	47	47	
**Vascular infiltration**					**<0.001**
No	284	85	190	58	
Yes	51	15	27	32	

NR, not reached

* Wedge, n = 10

Abbreviations: SCC; squamous cell carcinoma; LCC, large-cell carcinoma; BAC, bronchioalveolar carcinoma

### Interobserver variability

Interobserver scoring agreement was tested for miR-155. The scoring agreement was good (r = 0.91, P < 0.001).

### Expression of miR-155 and Correlations

MiR-155 was expressed in the cytoplasm of most neoplastic tumor cells and to a lesser extent expressed in the cytoplasm of normal epithelial cells in lung tissue. Based on morphological criteria, inflammatory cells (macrophages, lymphocytes, granulocytes and plasma cells), pneumocytes and fibroblasts, normal as well as tumor associated, showed variable and in general reduced cytoplasmic expression compared to tumor cells.

There were no significant correlations between miR-155 expression and any of the clinicopathological variables in the total material or in histological subgroups. There was a tendency (P = 0.076) towards higher frequency of high miR-155 expression in SCCs (52.4%) than ACs (40.4%). From our large database with expression data on different ligands, receptors and downstream proteins related to angiogenesis, hypoxia, epithelial-mesenchymal transition (EMT) as well as immunologic markers [23,26-33], the strongest association was found between miR-155 and phosphatase and tensin homologue (PTEN). There was an inverse correlation between miR-155 and PTEN expression, r = - 0.23, P < 0.001 (Table 2).

**Table 2:** Crosstab showing the inverse correlation between miR-155 and phosphatase and tensin homologue (PTEN).

		PTEN		Total
		**Low expression**	**High expression**	

**miR-155**	Low expression	119	40	159

	High expression	144	13	157

Total		263	53	316

Spearman correlation, r = - 0.23, P < 0.001

### Univariate Analysis

**Figure 2:**
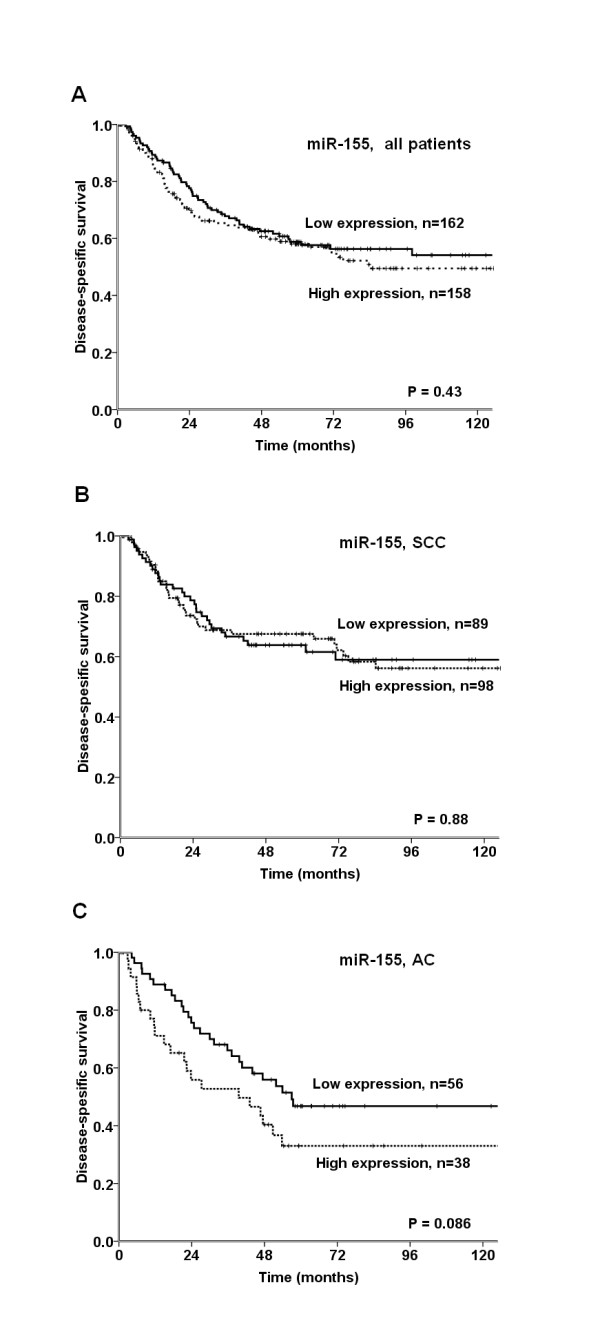
**Disease-specific survival curves according to miR-155 expression in: (A) the total material; (B) squamous cell carcinomas (SCCs); (C) adenocarcinomas (ACs)**.

**Figure 3:**
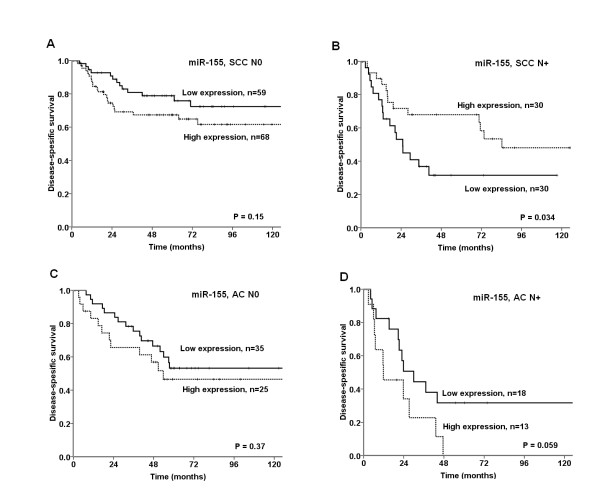
**Disease-specific survival curves according to histology and nodal status in NSCLC patients with: (A) squamous cell carcinomas (SCC) and negative lymph node status (N0); (B) SCC and positive lymph node status (N+); (C) adenocarcinomas (AC) and N0; (D) AC and N+**.

Survival analyses according to clinicophatological variables are shown Table 1. Performance status (P = 0.013), histology (P = 0.028), histological differentiation (P < 0.001), surgical procedure (P < 0.004), pathological stage (P < 0.001), T-stage (P < 0.001), N-stage (P < 0.001) and vascular infiltration (P < 0.001) were all significant prognostic indicators for DSS. DSS according to miR-155 expression is shown in Table 3 and Figure 2 and 3. In the total material (P = 0.43) and in the SCC subgroup (P = 0.88), miR-155 expression showed no significant prognostic impact. High miR-155 expression tended to a negative prognostic role in ACs (P = 0.086).

**Table 3:** Prognostic impact of miR-155 expression in the total material and histological and nodal status subgroups.

Characteristic	Pts (n)	Pts (%)	Median survival (months)	5-Year survival (%)	Uni-variate P	Multivariate P
**Total (n = 335)**					0.43	NS
Low	162	48	190	59		
High	158	47	84	58		
Missing	15	5				
**SCC (n = 191)**						NS
Low	89	47	133	64	0.88	
High	98	51	120	68		
Missing	4	2				
**SCC, N0**					0.15	NS
Low	59	47	160	79		
High	68	53	129	67		
**SCC, N+**					**0.034**	
Low	30	50	49	32		
High	30	50	95	68		HR 0.45, CI 95% 0.21-0.96, P = **0.039**
**AC (n = 95)**					0.086	
Low	56	62	104	47		
High	38	37	71	33		HR 1.87, CI 95% 1.01-3.48, P = **0.047**
Missing	1	1				
**AC, N0**					0.37	NS
Low	38	60	117	53		
High	25	40	93	47		
**AC, N+**					0.059	NS
Low	18	58	59	32		
High	13	42	20	0		

NS, not significant

In SCC patients with lymph node metastasis, high miR-155 expression appeared as a favorable prognostic factor (P = 0.034) while none of the clinicopathological variables were significant associated with DSS.

### Multivariate Cox Proportional Hazards Analysis

In the overall material, performance status (P = 0.008), histology (P = 0.001), pathological T-stage (P > 0.001), N-stage (P < 0.001), histological differentiation (P = 0.02) and vascular infiltration (P = 0.002) appeared as independent prognostic factors.

Results of miR-155 expression in multivariate analysis are presented in Table 3. For SCCs patients, N-stage (P = 0.001), histological differentiation (P = 0.011) and vascular infiltration (P = 0.037) were independent prognostic factors. In the SCC subgroup with nodal metastasis, high miR-155 expression was an independent significant positive prognostic factor (HR 0.45, CI 95% 0.21-0.96, P = 0.039) while none of the clinicopathological variables had independent prognostic impact.

For ACs patients, N-stage (P = 0.001), performance status (P = 0.001), vascular infiltration (P = 0.012) and miR-155 expression (HR 1.87, CI 95% 1.01 - 3.48, P = 0.047) were independent prognostic factors.

## Discussion

We present the first large-scale study combining high-throughput TMA and *in situ *hybridization to evaluate the prognostic impact of miR-155 expression. In this unselected population of surgically resected NSCLC patients, high miR-155 expression was an independent negative prognostic factor in ACs, while high miR-155 expression was an independent favorable prognosticator in SCC patients with regional nodal metastasis.

MiRNAs are well preserved in formalin-fixed tissue, making them attractive candidates for use in routinely processed material [34,35]. Most of the previous studies on miRNA expression were done on microarrays using RNA extracted from human cancer tissues samples and containing a mixture of neoplastic tumor cells and tumor related stromal cells. A major advantage of *in situ *hybridization is to precisely identify positive signals at the cellular level. For instance, recent data have demonstrated that some miRNAs had high expression levels in stromal cells but not in tumor cells [36]. Using RNA extracts from whole tumors, this finding would easily be missed.

Strengthening the relevance of our miR-155 expression data, there was a significant inverse correlation with PTEN. This corroborates a study by Yamanaka et al. showing that reduced expression of miR-155 led to up-regulation of PTEN in NK lymphoma cell lines [37].

Several studies have shown miR-155 to be overexpressed in NSCLC [13,14,16]. But, to our knowledge, only three studies have investigated the prognostic impact of miR-155 in NSCLC, all using quantitative RT-PCR as the principal method [13,16,18]. Yanaihara et al. [16], also using the median value as cut-off, found high miR-155 expression to be an independent negative prognostic factor in 64 stage I adenocarcinomas, corroborating our results.

Recently, Voortman et al. studied the prognostic and predictive values of a panel of miRs by quantitative real-time PCR in formalin-fixed paraffin-embedded tumor specimens from 639 resected NSCLC patients participating in the International Adjuvant Lung Cancer Trial (IALT) [18]. In the total cohort they found, consistent with our results, miR-155 to have no significant prognostic impact. However, subgroup analysis on the prognostic impact with regard to nodal status and histology was not reported. Raponi and coworkers identified 15 miRNAs that were differently expressed between epithelial cells in normal lung and stage I-III SCC, among them miR-155 [13]. Analysis of 54 SCC patients (63% N0) showed that high miR-155 expression tended to have a significant effect on survival (P = 0.06), while it was an unfavorable independent variable in multivariate analysis (HR 2.3, CI 95% 1.0 - 5.6). We found the same tendency (P = 0.15) in our N0 patients. More surprisingly, we found the opposite association in our SCC lymph node positive patients. This may indicate that the oncogenic miR-155 effect may become inhibited or overridden by other mechanisms in SCC patient with nodal metastasis. Though, as the number of cases in this subanalysis is limited (n = 30 in each arm) the result has to be interpreted carefully. There is always a danger of false positive results when stratifying in multiple subgroups. However, we have only stratified for histological classification and nodal status which are considered to be the two most important clinicopathological variables in NSCLC treatment strategies.

As an independent prognostic factor, miR-155 may be a relevant addition to clinicopathological variables in predicting outcome in adenocarcinoma patients. As a prognosticator, however, miR-155 expression appears more interesting in SCCs with nodal metastasis, as none of the clinicopathological variables were significant prognosticators in this subgroup. In the clinic, valid prognostic marker in the subpopulation of N+ patients is warranted and miR-155 seems to be a potentially interesting candidate, though further prospective validation studies are needed to confirm these results. Potential microRNA-based therapy is now being exploited in cancer, attempting to modulate their expression, reintroducing microRNAs lost in cancer, or inhibiting oncogenic microRNAs by using anti-micro oligonucleotides [38]. In a novel approach to inhibit microRNA function, synthetic mRNAs, called microRNA sponges, are able to bind up the microRNA, preventing its association with endogenous targets [39]. MiR-155 has also been suggested as a possible target in future treatment strategies. Indeed, as miR-155 (together with let-7a, miR-21 and miR17-92 cluster) is aberrantly expressed in a wide variety of hematological and solid malignancies, it has been speculated that strategies to silence miR-155 may have impact on multiple groups of cancer patients [40]. But according to our results, the miR-155 effect is apparently context specific, and though it may be relevant for a diversity of malignancies, an "individualized" approach is needed.

## Conclusion

MicroRNAs are well preserved in formalin-fixed tissue, making them ideal candidates for investigation in routinely processed material. Among the miRNAs, miR-155 is particularly interesting as it is consistently involved in several neoplastic diseases. By *in situ *hybridization we have been able to study cell specific expression of miR-155. Our results confirm that tumor cell miR-155 expression is a negative independent prognostic factor in adenocarcinomas. Further, we found high miR-155 expression to be a favorable independent prognostic factor in SCCs with lymph node metastasis. Further studies are needed to reveal the complexity of miR-155 function and, hopefully, the miR-155 status in various histological subtypes and stages of lung cancer may help to predict the toxicity and susceptibility to future RNA targeted therapies.

## Competing interests

The authors declare that they have no competing interests.

## Authors' contributions

TD participated in the design of the study, contributed to the clinical and demographic database, did the statistical analysis and drafted the manuscript. KE, TB and KL carried out and supervised the ISH. SWS and KE scored the cores. KAS, SAS, SA and HS contributed in the clinical and demographic database and KAS and SAS in making the TMAs. RB and LTB supervised and participated in the study design, result interpretation and in the writing.

All authors read and approved the final manuscript.
